# Robotic Thoracic Surgery After Neoadjuvant Chemo-Immunotherapy for NSCLC: A Narrative Review

**DOI:** 10.3390/cancers18142365

**Published:** 2026-07-22

**Authors:** Monica Casiraghi, Antonio Mazzella, Lara Girelli, Giorgio Lo Iacono, Luca Bertolaccini, Matteo Chiari, Giovanni Caffarena, Claudia Bardoni, Lorenzo Spaggiari

**Affiliations:** 1 Department of Thoracic Surgery, European Institute of Oncology (IEO) IRCCS, 20141 Milan, Italy; antonio.mazzella@ieo.it (A.M.); lara.girelli@ieo.it (L.G.); giorgio.loiacono@ieo.it (G.L.I.); luca.bertolaccini@ieo.it (L.B.); matteo.chiari@ieo.it (M.C.); giovanni.caffarena@ieo.it (G.C.); claudia.bardoni@ieo.it (C.B.); lorenzo.spaggiari@ieo.it (L.S.); 2 Department of Oncology and Hemato-Oncology, University of Milan, 20122 Milan, Italy

**Keywords:** chemo-immunotherapy, robotic surgery, NSCLC

## Abstract

**Background**: The integration of neoadjuvant and perioperative chemo-immunotherapy (CT-IO) has significantly reshaped the treatment of resectable non-small-cell lung cancer (NSCLC), improving pathological response and survival outcomes. However, its impact on surgical management—particularly robotic-assisted thoracic surgery (RATS)—remains incompletely defined. This review provides a practical overview of current evidence and technical considerations for robotic lung resection following neoadjuvant chemo-immunotherapy. **Methods**: A narrative review of the literature was performed, focusing on phase III trials, meta-analyses, and surgical series reporting perioperative, oncological, and technical outcomes of minimally invasive—especially robotic—approaches after neoadjuvant or perioperative chemo-immunotherapy. **Results**: Randomized trials have established CT-IO as a standard treatment option for selected patients with resectable stage II–III NSCLC—although the specific standard varies according to stage, molecular and PD-L1 status, and regulatory approval—significantly improving pathological complete response and event-free survival. However, immune-related fibrosis, nodal scarring, and altered tissue planes increase surgical complexity and intra-postoperative complications. Available evidence, largely retrospective and derived from selected patient populations treated at experienced centers, suggest that RATS is feasible and safe, offering enhanced visualization and dexterity that may facilitate dissection in challenging post-induction settings. Vascular management and lymph node dissection remain critical technical aspects, and early conversion to open surgery, when required, should be regarded as an appropriate safety strategy rather than a complication. **Conclusions**: RATS after neoadjuvant chemo-immunotherapy appears feasible and promising in selected patients treated at experienced centers, but current evidence does not yet establish it as the preferred approach for all patients. Careful patient selection, adherence to oncological principles, and surgeon experience are essential. Prospective data are needed to define optimal surgical timing and standardize techniques.

## Simple Summary

Neoadjuvant and perioperative chemo-immunotherapy (CT-IO) has become a standard treatment option for selected patients with resectable stage II–III non-small-cell lung cancer, improving pathological response and survival, although its effects on surgical management—particularly robotic-assisted thoracic surgery (RATS)—remain incompletely defined. This narrative review examined phase III trials and surgical series on minimally invasive approaches after CT-IO. Induction therapy increases surgical complexity through immune-related fibrosis and nodal scarring, but most supporting evidence for RATS in this setting is retrospective and derived from selected patients treated at experienced centers. Within these limits, RATS appears feasible and safe, with enhanced visualization and dexterity aiding dissection in challenging post-induction cases; early conversion to open surgery, when needed, should be regarded as an appropriate safety strategy rather than a treatment failure. Careful patient selection, adherence to oncological principles, and surgeon experience remain essential, and prospective studies are needed before RATS can be considered the preferred approach for all patients in this setting.

## 1. Introduction

The management of resectable non-small-cell lung cancer (NSCLC) has undergone a paradigm shift with the introduction of immune checkpoint inhibitors in the neoadjuvant and perioperative settings. Randomized phase III trials have demonstrated that the addition of immunotherapy (IO) to platinum-based chemotherapy (CT) significantly improves rates of major pathological response (MPR) and pathological complete response (pCR), as well as event-free survival (EFS) and overall survival (OS) [1,2,3,4,5].

More recently, perioperative strategies have further expanded the therapeutic landscape. Trials such as CheckMate 77T, Keynote-671, AEGEAN, NEOTORCH, and RATIONALE-315 evaluated approaches combining neoadjuvant CT-IO followed by adjuvant IO, and demonstrated significant improvements in pathological response and EFS compared with CT alone [2,3,4,6,7,8]. In parallel, the phase II NADIM II trial provided additional evidence supporting the efficacy of neoadjuvant nivolumab plus CT, reporting unprecedented rates of pCR and prolonged progression-free and OS in patients with resectable stage IIIA–IIIB disease [9]. While perioperative trials have shown benefits with the overall strategy, recent meta-analysis suggest that the incremental contribution of adjuvant IO after neoadjuvant CT-IO remains uncertain, with no significant additional EFS or OS benefit observed [10]. These advances have rapidly translated into clinical practice, establishing both neoadjuvant-only and perioperative CT-IO as new standards of care for selected patients with stage II–III NSCLC.

Despite these oncological benefits, the impact of IO on surgical resection remains an area of active investigation. Neoadjuvant treatment induces inflammatory and fibrotic changes within tumors and lymph nodes, often resulting in obliterated tissue planes and increased technical difficulty during surgery [11,12]. These challenges are particularly relevant for minimally invasive approaches.

Robotic-assisted thoracic surgery (RATS) offers potential advantages in this setting, including enhanced three-dimensional visualization, improved instrument articulation, and superior precision in confined anatomical spaces, facilitating complex hilar and mediastinal dissection in post-induction patients [13]. However, evidence on RATS following CT-IO remains limited, heterogeneous, and predominantly retrospective, and several key aspects—including patient selection, optimal surgical timing, technical approaches, and oncological adequacy—remain a matter of ongoing debate.

The increasing adoption of CT-IO has created a new surgical scenario in which excellent pathological responses frequently coexist with technically demanding resections. Understanding how IO-induced tissue changes affect minimally invasive surgery is becoming essential for thoracic surgeons.

The aim of this practical review is to summarize the current evidence regarding robotic lung resection after neoadjuvant CT-IO, such as surgical outcome and comparative evidence, and to provide practical recommendations on preoperative assessment, operative planning, and intraoperative management.

## 2. Materials and Methods

This narrative review was conducted to summarize current evidence regarding robotic-assisted thoracic surgery (RATS) after neoadjuvant and perioperative CT-IO for resectable NSCLC. A literature search was performed in PubMed, Embase, and Scopus for studies published between January 2018 and July 2026. Search terms included “NSCLC”, “neoadjuvant immunotherapy”, “chemo-immunotherapy”, “robotic thoracic surgery”, “robot-assisted thoracic surgery”, “RATS”, “video-assisted thoracic surgery”, and “lung resection”. Priority was given to phase II–III clinical trials, meta-analyses, multicenter studies, and comparative surgical series reporting perioperative, technical, or oncological outcomes. Additional references were identified through manual review of bibliographies. Studies were included if they reported original comparative or single-arm data on perioperative, technical, or oncological outcomes of lung resection (RATS, VATS, or open thoracotomy), specifically in patients who had received neoadjuvant or perioperative CT-IO for NSCLC; case reports, conference abstracts without a peer-reviewed full text, and studies that did not distinguish the surgical approach were excluded. Given the heterogeneity of the available evidence, a narrative rather than systematic approach was adopted, reflecting the substantial variability in study design, patient populations, and outcome definitions across the available series, most of which are small, single- or few-center retrospective cohorts; this heterogeneity would have limited the validity of formal quantitative pooling under a PRISMA-guided systematic framework. Consistent with this exploratory, narrative aim, no formal dual-reviewer screening process or PRISMA flow diagram was applied, and the number of records identified and excluded at each stage was not systematically tracked. The narrative format was also chosen to allow the integration of practical, technique-oriented recommendations (Section 4) alongside the comparative evidence summarized in Section 3.2, which falls outside the typical scope of a systematic review. We note that a narrative review with a technique- and technology-oriented focus [14] and a systematic review and meta-analysis restricted to RATS-versus-VATS comparisons [15] have been published on closely related topics; the present review differs in providing a structured, comparative synthesis of perioperative, conversion, and lymph node outcomes across MIS and open approaches, together with practical recommendations for preoperative, intraoperative, and postoperative management.

Generative artificial intelligence has been used in this paper to generate a simple summary, the abstract, tables, and graphics, and for superficial text editing.

## 3. Current Evidence

Recent key prospective trials have established neoadjuvant and perioperative CT-IO as new standards of care for selected patients with resectable NSCLC (Table 1).

CheckMate 816 was the first neoadjuvant-only IO regimen to show definitive OS benefit in resectable NSCLC, demonstrating that neoadjuvant-only nivolumab plus CT (without adjuvant IO) significantly improved OS compared with CT alone, with 5-year OS of 65.4% versus 55.0% [5]. Importantly, no patients who achieved pCR after neoadjuvant nivolumab plus CT in CheckMate 816 had reported death from lung cancer at 5 years, providing initial support for surveillance rather than further treatment in this subgroup [5].

In addition, Keynote-671 was the first perioperative trial to demonstrate definitive OS benefit (HR 0.72; 95% CI 0.56–0.93), while CheckMate 77T and AEGEAN extended this concept to perioperative strategies, including postoperative IO [2,3,4]. Additional evidence supporting the integration of immunotherapy into multimodal treatment strategies has emerged from several important phase II and phase III studies. The NADIM II trial demonstrated that neoadjuvant nivolumab combined with platinum-based CT significantly improved pCR rates (36.8% vs. 6.9%) and OS, compared with CT alone in patients with stage IIIA–IIIB NSCLC [9]. Similarly, the phase III NEOTORCH trial showed that perioperative toripalimab plus CT significantly improved EFS and pathological response, achieving a pCR rate of 24.8% compared with 1.0% in the CT-alone arm [6]. Consistent findings were also reported in the RATIONALE-315 study, where perioperative tislelizumab plus CT resulted in significantly higher pCR and MPR rates, further supporting the role of perioperative IO as a standard treatment option for resectable stage II–IIIA NSCLC [8].

Collectively, these studies reinforce the growing body of evidence supporting both neoadjuvant and perioperative IO-based strategies in resectable NSCLC. Beyond these trials, phase II studies and real-world surgical series have helped to clarify the practical implications of induction IO for thoracic surgeons.

### 3.1. Surgical Challenges After Neoadjuvant Chemo-Immunotherapy

From a surgical perspective, the growing efficacy of systemic therapy has not translated into simplification of the operation itself. Early post-IO reports showed technically demanding resections due to fibrosis and nodal desmoplasia [11]. Importantly, a discrepancy between pathological response and intraoperative difficulty has consistently been observed, as even patients achieving major or pCR may present with challenging hilar and mediastinal dissections [12]. A recurring observation across surgical series is the lack of correlation between pathological response and technical complexity. Patients achieving MPR or even pCR may still present extensive hilar fibrosis, nodal scarring, and difficult vascular dissection, leading to challenging surgery and higher risk of intra- and postoperative complications. Therefore, pathological response should not be interpreted as a surrogate marker of operative ease.

From a biological standpoint, the technical difficulty frequently observed after CT-IO is thought to reflect an immune-mediated desmoplastic reaction, characterized by dense fibro-inflammatory tissue, histiocytic and giant-cell infiltration, and occasional granuloma formation around lymph nodes and vascular structures. These changes can obliterate normal anatomical planes largely independently of the degree of tumor regression achieved, which explains why even patients with a major or complete pathological response may present with a technically hostile operative field, including calcified or matted nodes densely adherent to the pulmonary artery and bronchial wall. Awareness of this discordance between pathological response and operative difficulty is essential for surgical planning and patient counseling, as it cannot be reliably anticipated from preoperative cross-sectional imaging alone, underscoring the value of surgeon experience and setting a low threshold for conversion.

RATS after neoadjuvant CT-IO is feasible in selected patients, but surgeons should face a distinctly different operative field compared with upfront resection. Early surgical series after neoadjuvant IO and more recent robotic reports describe dense hilar fibrosis, vascular fragility, nodal scarring, and obliteration of tissue planes as the main technical challenges [11,12]. Radiological pseudoprogression and nodal immune flare—phenomena in which increased tumor or lymph node size on imaging reflects immune-cell infiltration rather than disease progression—can complicate preoperative staging and intraoperative decision-making, potentially requiring frozen-section guidance to differentiate immune response from residual disease [16,17].

These changes do not preclude minimally invasive resection, but they require careful planning, a low threshold for conversion, and substantial experience in advanced hilar dissection.

### 3.2. Surgical Outcomes and Comparative Evidence

Available surgical series and comparative studies provide important insights into the feasibility and technical implications of lung resection after neoadjuvant CT-IO (Table 2), in particular, in case of minimally invasive approaches such as RATS.

The available literature in this setting is characterized by substantial heterogeneity in study design, patient population, and reporting standards. Most series are retrospective and single-institutional, reflecting the experience of centers with established robotic thoracic surgery programs; this introduces a risk of selection and expertise bias that must be considered when interpreting comparative outcomes between RATS and VATS. Sample sizes range from small pilot cohorts of fewer than 50 patients to large multicenter or national database analyses including several thousand cases, and the proportion of patients with locally advanced (stage III) disease varies widely across studies, further limiting direct comparability. Direct comparative data restricted specifically to patients who received chemo-immunotherapy, as opposed to chemotherapy alone, remain particularly scarce, further constraining the strength of the conclusions that can currently be drawn regarding the specific impact of immunotherapy-related tissue changes on minimally invasive resection. Nonetheless, taken together, these data provide a reasonably consistent picture of feasibility across different healthcare systems and patient populations, spanning Western cohorts and large Asian multicenter series, and collectively support the incorporation of robotic surgery into the therapeutic algorithm for selected patients after CT-IO. Throughout this review, “selected patients” refers to those with favorable anatomical and clinical characteristics for a minimally invasive approach—including earlier clinical stage, smaller residual tumor size, absence of bulky or multi-station nodal disease, a favorable radiological or pathological response to induction therapy, and no anticipated need for extended or sleeve resection—operated on by surgeons and institutions with established experience in robotic thoracic surgery after induction therapy; the specific criteria informing this selection are detailed in Section 4.

**Table 2:** Surgical series and comparative studies on minimally invasive and robotic approaches after neoadjuvant CT-IO.

Study	*N*	Study Design	Neoadjuvant Regimen	Approach	Conversion Rate	Complications	Mortality	Key Findings
Bott et al. 2019 [11]	20	Phase I, single-arm (2 centers)	Nivolumab monotherapy × 2 doses	Mixed	54%	50%	0%	First series; hilar fibrosis challenging
Gao et al. 2022 [18]	44	Prospective, single-center	ICI + platinum doublet × 3 cycles	RATS	4.5%	11.4%	0%	RATS feasible stage III
Zhang et al. 2022 [19]	131	Retrospective, single-center	Chemo-immunotherapy (NR)	VATS vs. open	40.5% (VATS)	21.4%	0%	VATS fewer ICU stays
Deng et al. 2022 [20]	31	Retrospective, single-center	Immuno-chemotherapy (NR)	MIS	NR	16.1%	0%	Safe in unresectable IIIB
Zeng et al. 2023 [21]	220	Retrospective, single-center	PD-1/PD-L1 + platinum doublet × 3 cycles	RATS vs. VATS	7.5% vs. 28.2%	No difference	0%	RATS superior LN yield
Pan et al. 2023 [22]	46	Retrospective, single-center	Chemo-immunotherapy (NR)	RATS vs. VATS	NR	No difference	RATS 0% vs. VATS 3.2%	RATS reduced ICU stay
Li HJ et al. 2025 [23]	435	Retrospective, PSM	NR	RATS vs. VATS	8.5% vs. 18.6%	NR	NR	RATS shorter operative time
Li X et al. 2025 [24]	369	Retrospective, PSM	NR	RATS vs. VATS	3.1% vs. 8.9%	Air leak: 1.6% vs. 7.7%	NR	RATS superior sleeve lobectomy
Kneuertz et al. 2025 [17]	207	Retrospective, multicenter (5 centers)	Chemo-immunotherapy (NR)	MIS vs. open	4.5% vs. 31% (*RATS* vs. *VATS*)	9.1% vs. 25.6%	1%	pCR predicts MIS success (OR 10.81)
Pan et al. 2025 [25]	571	Retrospective, multicenter (12 centers)	Immuno-chemotherapy (NR)	MIS vs. open	15.8%	MIS fewer	NR	Nomogram for conversion (AUC 0.804)
Pohlman et al. 2026 [13]	3937	Retrospective, national database	NR	RATS vs. VATS	7.9% vs. 17.2%	NR	NR	VATS 2× odds conversion (aOR 1.98)
Bertoglio et al. 2026 [26]	2691	Systematic review/meta-analysis	N/A (pooled)	Meta-analysis	20% (pooled)	27% (pooled)	1% (pooled)	MIS 47%; safe overall

RATS = robotic-assisted thoracic surgery; VATS = video-assisted thoracic surgery; MIS = minimally invasive surgery; LN = lymph node; pCR = pathological complete response; NR = not reported; OR = odds ratio; aOR = adjusted odds ratio.

Early reports, such as the experience described by Bott et al., demonstrated that pulmonary resection after induction IO is still feasible, although often associated with dense hilar fibrosis and inflammatory changes that may significantly increase operative complexity [11]. Subsequent prospective analyses, including data from the NEOSTAR platform, confirmed that perioperative outcomes are generally acceptable, but also highlighted a key discrepancy between pathological response and intraoperative difficulty. Indeed, approximately 52.3% of cases were considered more challenging than a standard lobectomy, with 59.1% rated as challenging in the nivolumab plus CT arm, even among patients achieving major or complete pathological response [12].

More recent retrospective series have further supported the feasibility of minimally invasive approaches after CT-IO [22,25,26,27,28,29,30]. Deng et al. reported acceptable morbidity and low perioperative mortality in patients undergoing resection after IO, reinforcing the safety of surgery in this setting when performed in experienced centers [20]. However, these studies also emphasize that patient selection remains critical, as technical complexity may vary widely during MIS, in particular when considering different approaches such as RATS and VATS. Multiple studies suggest that RATS may offer advantages in terms of short-term outcomes and technical handling in post-induction cases, likely due to enhanced visualization and instrument articulation [25,26,27].

#### 3.2.1. Perioperative Outcomes

RATS has been associated with reduced blood loss, shorter operative time, shorter chest tube duration, and lower rates of persistent air leak (>7 days: 1.6% vs. 7.7%, *p* = 0.017) compared to VATS [26]. These benefits appear particularly pronounced in complex resections such as sleeve lobectomy, where RATS further reduced operative time and chest tube duration [26].

These findings are broadly consistent across several retrospective series comparing robotic and video-assisted approaches after induction therapy. Gao et al. reported a low conversion rate (4.5%) among 44 patients undergoing RATS after neoadjuvant chemo-immunotherapy for locally advanced disease [18], supporting the feasibility of the robotic approach even in more advanced clinical stages, with an 11.4% complication rate and no perioperative mortality. Conversely, Zhang et al., in a mixed cohort of 131 patients comparing VATS with open thoracotomy, observed a markedly lower conversion rate with the minimally invasive approach (21.4% versus 40.5%) [19], together with fewer intensive care unit admissions, although this series did not directly evaluate a robotic arm. Taken together, these data suggest that the three-dimensional visualization, wristed instrumentation, and improved ergonomics of robotic platforms may translate into a measurable, if modest, perioperative advantage in the post-induction setting compared with conventional VATS, particularly for technically demanding dissections such as sleeve resections, where reduced operative time and shorter chest tube duration have been specifically reported [16,17]. However, most of these comparisons are retrospective, non-randomized, and unmatched for baseline tumor stage, comorbidities, and surgeon experience; consequently, the true magnitude of the robotic advantage over VATS should be interpreted with appropriate caution pending prospective, adequately powered validation.

A recent systematic review and meta-analysis of 27 prospective trials (*n* = 2691) reported pooled postoperative complication rates of 27%, postoperative mortality of 1%, and minimally invasive surgery utilization of 47%, with a conversion rate of 20% [29]. Another meta-analysis of 15 studies found a pooled surgical resection rate of 98.9% with 16.5% of conversion rate associated [30]. These data confirm that surgery after neoadjuvant CT-IO is safe and feasible, with outcomes comparable to historical surgical benchmarks when performed in experienced centers.

#### 3.2.2. Predictors of Conversion

One of the largest national analyses of 3937 patients, undergoing minimally invasive lobectomy after neoadjuvant therapy, found that VATS was independently associated with nearly twice the odds of conversion to open thoracotomy compared to RATS (adjusted OR 1.98, *p* = 0.001), with overall conversion rates of 17.2% for VATS versus 7.9% for robotic approaches [13]. A multicenter study of 207 patients undergoing minimally invasive or open resection similarly reported an overall unplanned conversion rate of 9.1%, which was significantly lower with a robotic-assisted approach than with VATS within the minimally invasive cohort (4.5% vs. 31%, *p* = 0.001) [17].

From these data, a nomogram was developed with good performance (AUC 0.804) for preoperative prediction of conversion probability. Importantly, pCR was an independent predictor of successful minimally invasive surgery (OR 10.81; 95% CI 2.71–43.20; *p* = 0.001); this is a postoperative association rather than a preoperative selection criterion, since pCR is confirmed only after resection, but it indicates that patients who ultimately achieve a complete pathological response tend to have undergone a less technically demanding minimally invasive procedure [17]. Another multicenter study with more than 500 patients analyzed (*n* = 571) identified independent risk factors for conversion from MIS to open thoracotomy, such as squamous cell histology, advanced pre-induction N stage, preoperative tumor size > 5 cm, advanced preoperative stage, and need for extended resection [28].

Collectively, these predictive factors reinforce the concept that anatomical and biological variables—rather than the surgical approach alone—largely determine the technical complexity of resection after CT-IO. Squamous histology, in particular, has repeatedly emerged as a risk factor across independent cohorts, possibly reflecting a greater propensity for central, hilar-based tumors, and more pronounced peribronchial inflammatory change compared with adenocarcinoma. Surgeon and institutional experience with robotic thoracic surgery likely modulates the impact of these risk factors, and the learning curve required to master robotic dissection in fibrotic, post-induction fields should not be underestimated when extrapolating these findings to lower-volume centers. Incorporating such variables into preoperative counseling may help set realistic expectations regarding operative time, blood loss, and the likelihood of conversion, and may support a more individualized choice between robotic, video-assisted, and open approaches at the outset, rather than reactively during surgery. The development of externally validated risk-prediction tools, such as the nomogram proposed by Pan et al. [25], with good discriminative performance (AUC 0.804), represents an important step toward standardizing patient selection across institutions with differing levels of experience in minimally invasive thoracic surgery, although external validation in independent, geographically diverse cohorts remains necessary before such tools can be broadly recommended for routine clinical use.

#### 3.2.3. Lymph Node Assessment

RATS has demonstrated superior LN dissection compared to VATS. Zeng et al. reported that RATS yielded significantly more LN node assessment after induction treatments [21]. In particular, Pan et al. found that RATS assessed more N1 lymph nodes and stations compare to other minimally invasive approach [22]. This oncological adequacy is particularly important given the prognostic significance of accurate nodal staging after neoadjuvant therapy.

Beyond the absolute number of lymph nodes retrieved, the completeness and thoroughness of nodal dissection carry direct oncological relevance in the post-induction setting, since accurate pathological nodal staging informs decisions regarding adjuvant therapy, prognosis, and long-term surveillance strategy. The robotic platforms may facilitate a more systematic and complete exploration of nodal stations that are often most affected by treatment-related fibrosis, such as stations 4, 7, 10 and 11, where lymph nodes are frequently adherent to the airway, esophagus, and pulmonary vasculature. This potential advantage is particularly relevant given that inadequate nodal sampling after neoadjuvant therapy may lead to understaging and, consequently, to missed opportunities for adjuvant treatment. Nonetheless, differences in the definition of adequate nodal dissection—whether expressed as total node count, number of stations sampled, or systematic mediastinal lymphadenectomy—limit direct comparability across studies, and future series should adopt standardized, IASLC-consistent reporting of station-specific yield to allow meaningful pooled analyses.

#### 3.2.4. Limitations of Current Evidence

Overall, the evidence supporting robotic surgery after neoadjuvant CT-IO remains predominantly retrospective, drawn largely from single-center experiences or national database analyses rather than randomized comparisons. Sample sizes are generally modest, follow-up is often limited to short-term perioperative endpoints rather than long-term oncological outcomes, and definitions of conversion, complication severity, and adequate lymphadenectomy vary substantially between studies, complicating direct comparison and meaningful meta-analytic pooling. Furthermore, most series originate from high-volume academic centers with established, mature robotic programs, which may not reflect outcomes achievable in lower-volume or less experienced settings, introducing an important generalizability concern, and publication bias toward favorable results cannot be excluded. Cost-effectiveness analyses comparing robotic and video-assisted platforms in this specific population are also lacking, an important consideration given the higher upfront costs associated with robotic surgery. These limitations underscore the need for prospective, multicenter studies with standardized outcome definitions and, ideally, direct head-to-head comparison of RATS, VATS, and open surgery specifically in the post-CT-IO population, to confirm whether the perioperative and oncological advantages suggested by retrospective data translate into a genuine and reproducible clinical benefit.

## 4. Practical Surgical Considerations

An expert survey data, published by Wu in 2025, regarding the influence of neoadjuvant IO on surgical approaches in NSCLC, suggest that 81.2% of thoracic surgeons prefer a 4–6 weeks window after neoadjuvant IO, which aligns with the typical 3–6 weeks interval used in the phase III trials [18]. A multicenter retrospective study found that surgery performed within 4–6 weeks after the completion of neoadjuvant CT-IO was associated with improved pCR rates and disease-free survival compared to shorter (4 weeks) or longer (>6 weeks) intervals [19]. However, emphasis should be placed on early surgical treatment after neoadjuvant therapy, particularly in patients who have poor or no response to treatment. A practical checklist for surgeons managing patients undergoing RATS after neoadjuvant chemo-immunotherapy (Table 3).

### 4.1. Preoperative Assessment

Accurate restaging following neoadjuvant CT-IO is critical before surgical planning. Persistent radiological abnormalities after treatment do not necessarily correspond to viable tumor, as immune-cell infiltration may lead to pseudoprogression or nodal immune flare [31]. Consequently, radiological findings should be interpreted with caution and integrated with clinical assessment and, when indicated, invasive mediastinal staging.

Patient selection remains a cornerstone of successful MIS. Tumors larger than 5 cm, bulky hilar disease, advanced nodal involvement, and the anticipated need for extended resections have been associated with increased risk of conversion to thoracotomy. Conversely, patients achieving major pathological response or pathological complete response appear to be particularly suitable candidates for minimally invasive approaches.

However, pathological response is confirmed only postoperatively and cannot itself serve as a preoperative selection criterion. Preoperatively, restaging imaging can at most demonstrate a radiological complete response, which may correlate with, but is not a reliable substitute for, a pathological complete response; this finding may therefore suggest, but should never replace, intraoperative judgment regarding the feasibility of a minimally invasive approach.

### 4.2. Operative Planning

Preoperative imaging should be reviewed carefully to identify possible vascular encasement, fused hilar LN, and post-treatment anatomical distortions. Surgeons should anticipate more difficult hilar dissection than suggested by imaging findings alone. When a robotic approach is planned, availability of vascular control instruments and a predefined conversion strategy are essential. The operating team should be prepared for rapid thoracotomy in case of major vascular injury.

### 4.3. Intraoperative Management

The most frequent technical challenges after CT-IO include dense hilar fibrosis, obliteration of tissue planes, adherent or calcified lymph nodes, pulmonary artery fragility, and mediastinal scarring [32]. Robotic technology may facilitate dissection; however, oncological principles should never be compromised in an attempt to preserve a minimally invasive approach. Particular attention should be paid to station 10 and 11 LN, which are frequently fused with vascular structures after treatment. In these situations, proximal vascular control should be obtained early, and conversion should not be considered a failure but rather a strategy to ensure patient safety.

Conversion should be considered whenever safe vascular dissection cannot be achieved, when visualization becomes inadequate, or when oncological radicality is at risk.

**Table 3:** Practical checklist for surgeons managing patients undergoing RATS after neoadjuvant chemo-immunotherapy.

Phase	Checklist Item
Preoperative	Review restaging imaging with radiology; distinguish pseudoprogression/nodal immune flare from viable disease
Preoperative	Consider invasive mediastinal staging if restaging findings are ambiguous
Preoperative	Assess risk factors for conversion (tumor > 5 cm, bulky N2, squamous histology, anticipated extended resection)
Preoperative	Plan timing of surgery (target 4–6 weeks after completion of neoadjuvant therapy)
Operative planning	Ensure availability of vascular control instruments before starting
Operative planning	Define a predefined conversion strategy in advance
Operative planning	Confirm surgeon/team experience with robotic dissection in fibrotic, post-induction fields
Intraoperative	Anticipate dense hilar fibrosis, PA fragility, and fused lymph nodes (stations 10/11)
Intraoperative	Obtain proximal vascular control early when dissecting fused nodes
Intraoperative	Maintain a low threshold for conversion; treat it as a safety strategy, not a failure
Postoperative	Confirm R0 resection and adequate nodal staging
Postoperative	Discuss adjuvant therapy in a multidisciplinary setting based on pathological response

## 5. Future Perspectives

The rapid evolution of perioperative IO is expected to further influence the surgical management of resectable NSCLC. Several unanswered questions remain, including the optimal timing of surgery after induction therapy, the role of surgery in patients achieving radiological or complete response, and the identification of biomarkers capable of predicting pathological response and operative complexity.

Circulating tumor DNA (ctDNA), radiomics, and artificial intelligence-assisted imaging analyses may improve patient selection and response assessment in the near future [33,34,35]. Furthermore, increasing the adoption of robotic platforms may facilitate complex resections following induction therapy through improved visualization, instrument dexterity, and integration of advanced imaging technologies.

Future prospective studies should focus not only on oncological outcomes but also on technical endpoints, including conversion rates, vascular complications, LN assessment, and quality of surgical resection. Standardization of surgical strategies in the post-IO setting will be essential to optimize outcomes and facilitate comparisons across institutions.

Beyond their diagnostic values in isolation, these emerging tools could be integrated directly into surgical decision-making. Post-treatment ctDNA clearance or persistence could help distinguish true residual viable tumor from radiological pseudoprogression or nodal immune flare, complementing the intraoperative frozen-section assessment discussed above. Radiomic signatures derived from post-induction imaging could contribute to estimating the risk of conversion or of a technically difficult dissection before the patient enters the operating room, supporting the choice between RATS, VATS, and planned open surgery, as discussed in Section 4. AI-assisted, longitudinal analysis of sequential CT scans may likewise help select patients most likely to benefit from a minimally invasive approach and flag those for whom a lower threshold for early conversion is advisable. Prospective validation of these tools, ideally embedded within multicenter surgical cohorts rather than developed in isolation, will be necessary before they can be incorporated into routine preoperative planning.

## 6. Conclusions

Neoadjuvant and perioperative CT-IO have transformed the treatment landscape of resectable NSCLC, and are increasingly encountered in daily thoracic surgical practice. Although these strategies improve pathological response and survival outcomes, they frequently induce inflammatory and fibrotic changes that increase operative complexity. Within selected patients and experienced centers, RATS appears feasible and safe, offering potential advantages thanks to better visualization and dexterity in lymph node and hilar dissection; when a minimally invasive approach becomes unsafe, early conversion to thoracotomy should be regarded as a deliberate safety strategy rather than a failure. Nevertheless, current evidence does not yet establish RATS as the preferred approach for all patients, and successful implementation requires careful patient selection, meticulous operative planning, and surgeon experience.

**Figure 1:**
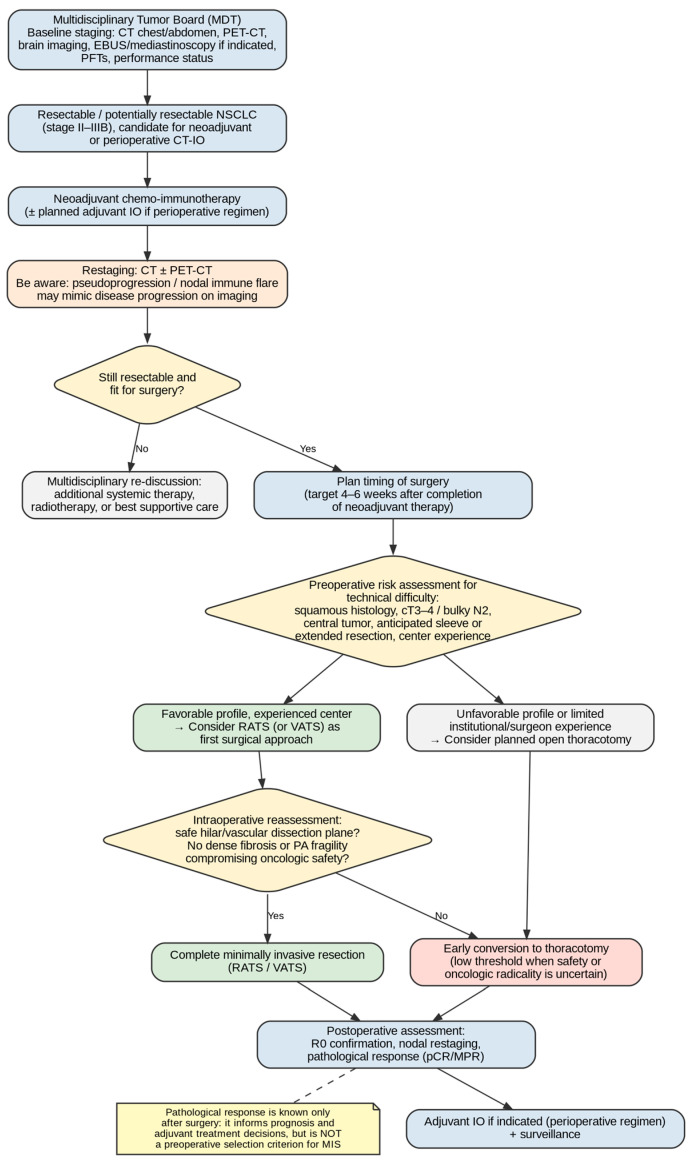
Proposed multidisciplinary decision-making algorithm for the surgical management of resectable non-small-cell lung cancer (NSCLC) after neoadjuvant or perioperative chemo-immunotherapy (CT-IO), from baseline staging through postoperative management. MIS: minimally invasive surgery; RATS: robotic-assisted thoracic surgery; VATS: video-assisted thoracoscopic surgery; PA: pulmonary artery; pCR: pathological complete response; MPR: major pathological response.

Current evidence supporting robotic surgery in this setting remains largely retrospective and derived from selected populations at high-volume centers. Beyond summarizing recent trials and surgical series, this review contributes a structured synthesis of the comparative perioperative and technical evidence for RATS after CT-IO, a practical decision-making algorithm (Figure 1) integrating oncological, radiological, and surgical considerations, and a set of concrete recommendations for preoperative assessment, operative planning, and intraoperative management (Section 4). Prospective studies remain needed to define optimal patient selection criteria, establish standardized technical approaches, and clarify the role of robotic surgery within the evolving multidisciplinary management of resectable NSCLC.

## Figures and Tables

**Table 1:** Key prospective trials of neoadjuvant and perioperative CT-IO in resectable NSCLC.

Trial	*N*	Stage	Strategy	ICI Regimen	pCR	MPR	EFS HR (95% CI)	OS HR (95% CI)
CheckMate 816	358	IB–IIIA	Neoadjuvant	Nivolumab × 3	24.0% vs. 2.2%	36.9% vs. 8.9%	0.63 (0.43–0.91)	0.72 (0.52–1.00)
KEYNOTE-671	797	II–IIIB	Perioperative	Pembrolizumab × 4 + adj	18.1% vs. 4.0%	30.2% vs. 11.0%	0.58 (0.46–0.72)	0.72 (0.56–0.93)
CheckMate 77T	461	IIA–IIIB	Perioperative	Nivolumab × 4 + adj	25.3% vs. 4.7%	35.4% vs. 12.1%	0.58 (0.42–0.81)	0.85 (0.58–1.25) *
AEGEAN	802	II–IIIB	Perioperative	Durvalumab × 4 + adj	17.2% vs. 4.3%	33.3% vs. 12.3%	0.68 (0.53–0.88)	0.89 (0.70–1.14) *
NADIM II	86	IIIA–IIIB	Perioperative	Nivolumab × 3 + adj	36.8% vs. 6.9%	52.6% vs. 27.6%	NR	NR
Neotorch	404	III	Perioperative	Toripalimab × 3 + adj	24.8% vs. 1.0%	48.5% vs. 8.4%	0.40 (0.28–0.57)	0.62 (0.38–1.00) *
RATIONALE315	453	II–IIIA	Perioperative	Tislelizumab × 4 + adj	24.5% vs. 6.2%	46.2% vs. 14.2%	0.56 (0.40–0.79)	NR

ICI = immune checkpoint inhibitor; adj = adjuvant immunotherapy; pCR = pathological complete response; MPR = major pathological response; EFS = event-free survival; OS = overall survival; HR = hazard ratio; CI = confidence interval; NR = not reported. * OS data are immature or not formally tested at the time of the published analysis.

## Data Availability

The original contributions presented in this study are included in the article.

## Abbreviations

The following abbreviations are used in this manuscript:

NSCLC	Non-Small-Cell Lung Cancer
CT	Chemotherapy
IO	Immunotherapy
MPR	Major Pathological Response
pCR	Pathological Complete Response
RATS	Robotic-Assisted Thoracic Surgery
EFS	Event-Free Survival
OS	Overall Survival
VATS	Video-Assisted Thoracic Surgery
MIS	Minimally Invasive Surgery
LN	Lymph Node
